# Trends in Cancer Incidence in Maputo, Mozambique, 1991–2008

**DOI:** 10.1371/journal.pone.0130469

**Published:** 2015-06-25

**Authors:** Cesaltina Lorenzoni, Alba Vilajeliu, Carla Carrilho, Mamudo R. Ismail, Paola Castillo, Orvalho Augusto, Alberto L. García-Basteiro, Mohsin Sidat, Silvia de Sanjosé, Clara Menéndez, Jaume Ordi

**Affiliations:** 1 Department of Pathology, Maputo Central Hospital, Maputo, Mozambique; 2 Faculty of Medicine, Eduardo Mondlane University, Maputo, Mozambique; 3 ISGlobal, Barcelona Ctr. Int. Health Res. (CRESIB), Hospital Clínic—Universitat de Barcelona, Barcelona, Spain; 4 Department of Preventive Medicine and Epidemiology, Hospital Clinic, Universitat de Barcelona, Barcelona, Spain; 5 Department of Microbiology, Faculty of Medicine, Eduardo Mondlane University, Maputo, Mozambique; 6 Unit of Infections and Cancer, Institut Catala d'Oncologia, L'Hospitalet de Llobregat, Barcelona, Spain; 7 Centro de Investigação em Saúde de Manhiça (CISM), Maputo, Mozambique; 8 Department of Pathology, Hospital Clinic, Universitat de Barcelona, Barcelona, Spain; Wayne State University School of Medicine, UNITED STATES

## Abstract

**Background:**

Very limited information is available regarding the incidence of cancer in sub-Saharan Africa. We analyzed changes in cancer patterns from 1991 to 2008 in Maputo (Mozambique).

**Methods:**

We calculated the rates of incidence of different cancer sites by sex in the 5-year age-group of the population of Maputo city as well as age-standardized rates (ASRs) and average annual percentage changes (AAPC).

**Results:**

Over the 18-year study period a total of 12,674 cases of cancer (56.9% females) were registered with an overall increase in the risk of cancer in both sexes. In males, the most common cancers were those of the prostate, Kaposi sarcoma (KS) and the liver. Prostate cancer showed the most dramatic increase over the whole study period (AAPC +11.3%; 95% CI: 9.7–13.0), with an ASR of 61.7 per 10^5^ in 2003–2008. In females, the most frequent cancers were of the uterine cervix, the breast and KS, with the former increasing along the whole study period (AAPC + 4.7%; 95% CI: 3.4–6) with an ASR of 62.0 per 10^5^ in 2003–2008 as well as breast cancer (AAPC +6.5%; 95%CI: 4.3–8.7).

**Conclusions:**

Overall, the risk of cancer rose in both sexes during the study period, particularly among cancers associated with westernization of lifestyles (prostate, breast), combined with increasingly rising incidences or limited changes in cancers associated with infection and poverty (uterine cervix, liver). Moreover, the burden of AIDS-associated cancers has shown a marked increase.

## Introduction

Knowledge of cancer patterns in the different populations is crucial to guide preventive efforts [1–4]. For this reason, cancer registries have been developed to record epidemiological data that are used to guide cancer prevention and control programs [1–5]. Unfortunately, very limited information is available regarding the incidence of cancer in sub-Saharan Africa[1,4–6]. This may be due to different reasons including limited resources in collecting and registering information as well as the lack of stability in maintaining registries over long periods of time. All of this has precluded evidence-based analysis of time patterns related to the incidence of cancer in this region[4,7,8].

After the end of the civil war in 1991 the Department of Pathology of the Maputo Central Hospital (MCH) started a cancer registry with the aim of obtaining information on the occurrence of cancer in the population of Maputo city. This lengthy registration of data has provided a unique opportunity to study temporal trends in cancer patterns in an African setting.

Interestingly, epidemiological data had previously been obtained by the Department of Pathology of the MCH (then known as Miguel Bombarda Hospital) from 1956 to 1961 in a cancer survey covering the metropolitan area of 60 square kilometers of Maputo (then known as Lourenço Marques) [9].

More than 50 years later, significant social and epidemiological changes have taken place in Mozambique, with a progressive urbanization of the population and the eruption of the HIV epidemic which has disproportionately affected many sub-Saharan African countries[9].

In the present study we analyzed the changes in cancer patterns in Maputo city, Mozambique from 1991 to 2008.

## Materials and Methods

### Study Site

This study was performed in the Department of Pathology of the MCH, a 1500-bed hospital that is the only quaternary care center in Mozambique and is a national referral center. This department primarily serves the Southern Zone of Mozambique including Maputo and its surroundings (provinces of Maputo, Gaza and Inhambane), with a small proportion of cases derived from other areas of the country due to the national referral nature of the institution. The department receives virtually all specimens from the city of Maputo. The department has a database of all the samples received for pathological diagnosis including specimens of most types of cancers (with the exception of leukemia and cancers with only a clinical diagnosis).

### Study Design

This study was approved by the National Bioethics Committee of Mozambique, and the Mozambican Ministry of Health (Ref. 389/CNBS). The analysis included all the cases of cancer registered in the Department of Pathology of the MCH from January 1, 1991 to December 31, 2008. Data were entered into a Microsoft Access database (Microsoft Co, Redmond, WA, USA) which, upon data entry, prevents the use of nonexistent codes and performs checks for internal consistency between variables. The database was carefully reviewed (name, age) to confirm that no duplications had been registered. Information about previous analyses in the same patient was also obtained, in order to identify potential duplicate registrations. Cases were identified in the registries as histological studies (including biopsies and surgical pathology), cytological specimens, and autopsies. For each case identified basic demographic data were collected, including age, sex and address. Patients from sites other than Maputo were excluded. For each specimen, the date of diagnosis, method of diagnosis, site from which the specimen was taken (topography), and microscopic morphology were recorded. Patient records/information were anonymized and de-identified prior to analysis. All topographic and morphologic classifications were made according to the International Classification of Diseases for Oncology (ICD-O)[10,11] and were converted into the 10^th^ version of the ICD[12]. In concordance with other cancer registries, cancers such as Kaposi’s sarcoma and malignant lymphomas were not classified by localization, as they are considered multifocal, and were included only once in the analysis[5,7]. Multiple cancers occurring in the same patient were entered as separate cases. Squamous cell carcinomas (SCCs) of the conjunctiva were defined as tumors with ICD-O morphology codes M8010–M8082 of conjunctiva ICD-O C69.0) or eye, unspecified (ICD-O C69.9).

### Population

**Fig 1:**
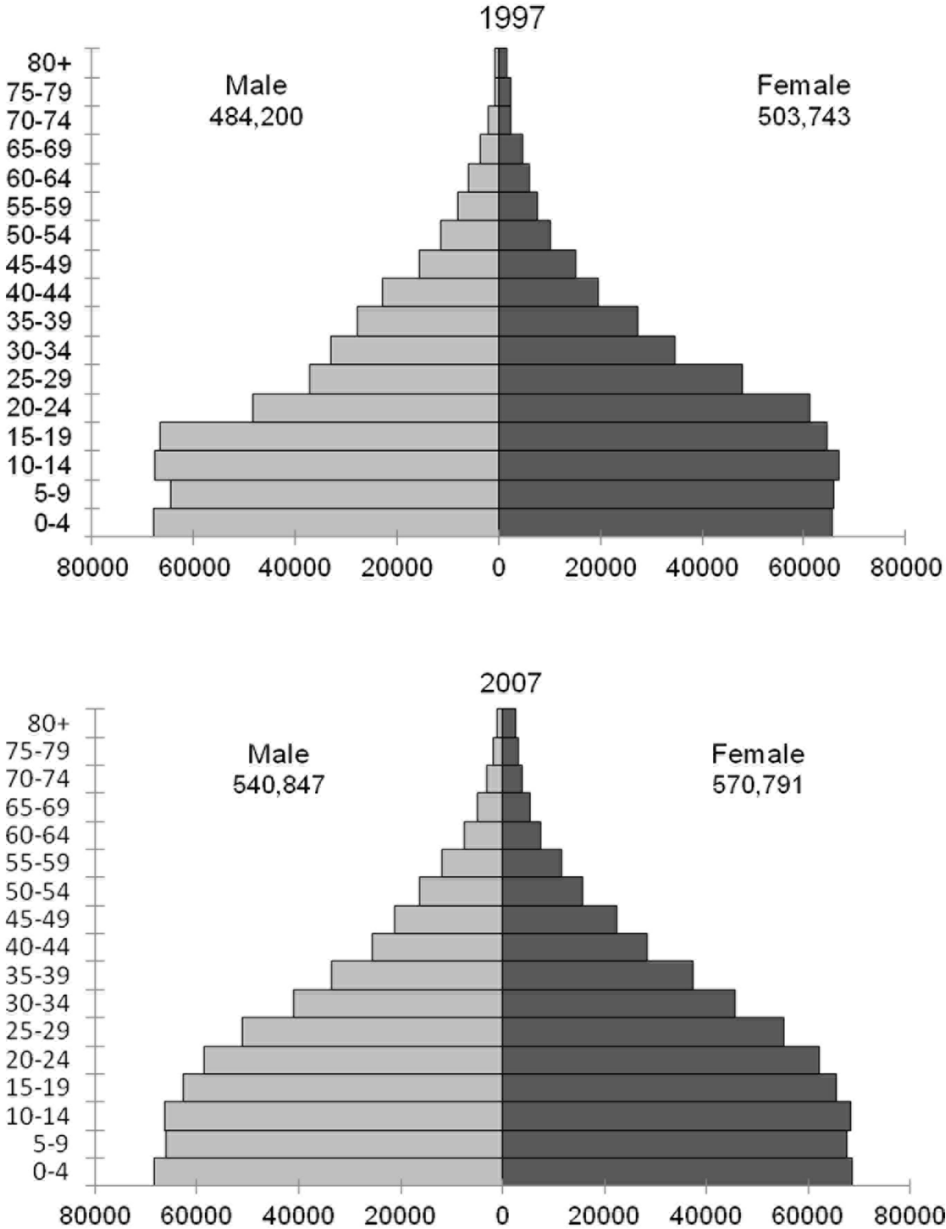
Population of Maputo by gender and age groups in the censuses of 1997 and 2007.

Censuses of the population of Mozambique were performed in 1980[13], 1997[14] and 2007[15], providing demographic information of Maputo by sex and 1 year of age groups for these years. Intercensal estimates were calculated assuming a constant rate of change in growth within sex and age groups. Fig 1 shows the population of Maputo by gender and age groups in the censuses of 1997 and 2007.

### Statistical Analysis

The rates of incidence of cancer were calculated for the 5-year age-group by sex, for each year (1991–2008) and for three time periods: 1991–1996, 1997–2002 and 2003–2008. Age-standardized rates (ASRs) were calculated using the world standard population[16]. The average annual percentage change (AAPC) and 95% confidence intervals (95%CI) over the 18-year study period were calculated for each sex and cancer sites. The AAPC was estimated on fitting a Poisson regression model to the natural logarithm of the counts offset for the logarithm of the person-years, including the calendar period as a continuous variable to estimate the slope. The calculation of the AAPC assumes that the rates increase or decrease log-linearly over the entire period[17,18]. Graphs on time trend estimates show 3-year moving average values of rates to minimize fluctuation due to small numbers of cases.

## Results

**Fig 2:**
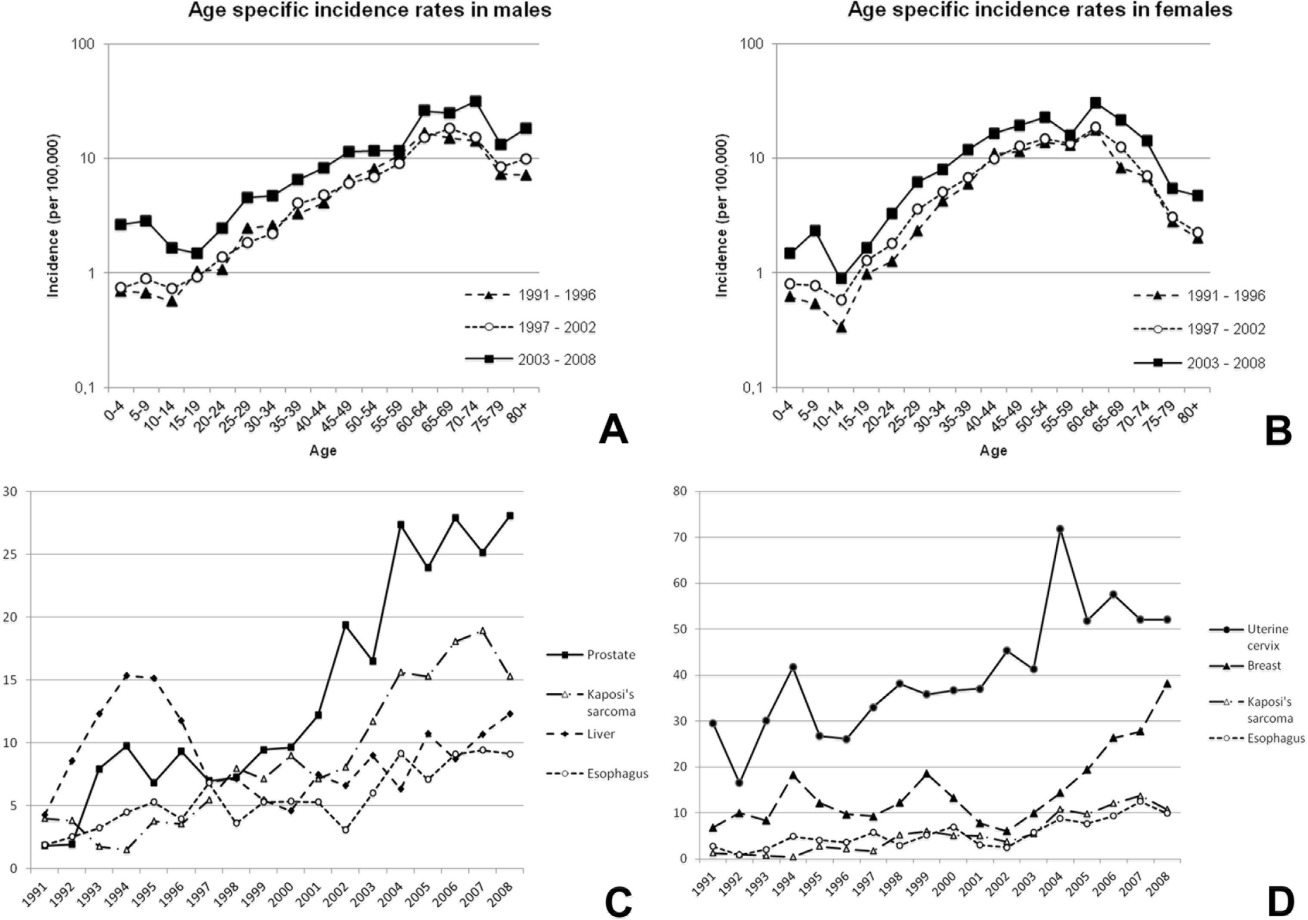
Age-specific incidence rates of male (A) and female (B) cancers in the periods 1991–1996, 1996–2001 and 2003–2008; and trends in age-standardized incidence rates of the four most frequent cancers in males (C) and females (D) from 1991–2008.

**Fig 3:**
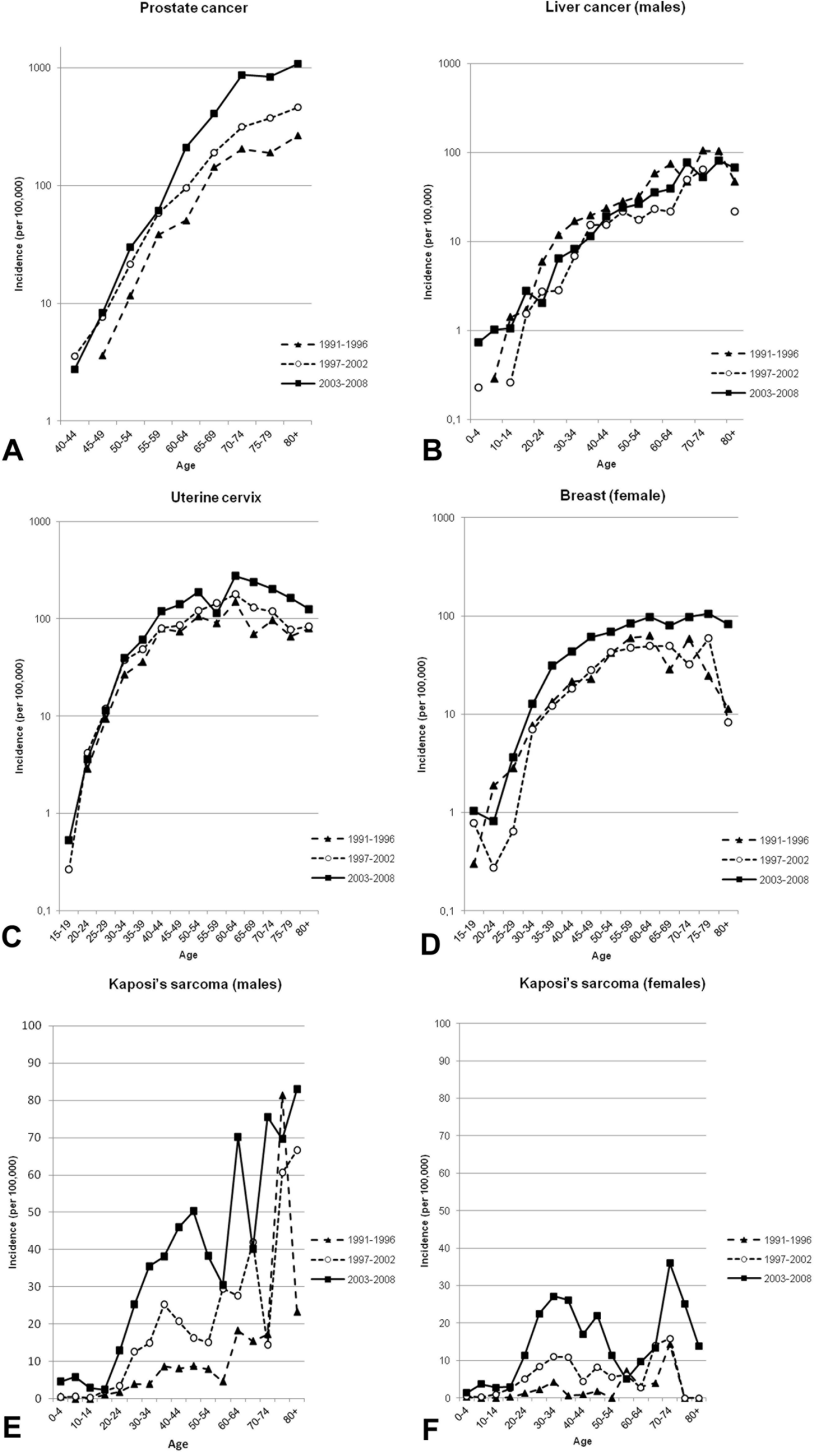
Age-specific incidence rates of relevant cancers in the periods 1991–1996, 1996–2001 and 2003–2008. A) prostate; B) liver (males); C) uterine cervix; D) breast (females); and Kaposi’s sarcoma in males (E) and females (F).

During the 18 years of registration included in the analysis (1991–2008) a total of 12 674 cases (5415 males and 7208 females, sex not recorded in 51) were registered. The rates of the incidence of cancer were shown to have increased in both sexes over the study period with an AAPC of +4.8% (95% CI: 4.0–5.5) for males and +5.1% (95% CI: 4.3–5.9) for females. The ASR for the 1991–1996 period was 102.3 per 10^5^ in males and 102.6 per 10^5^ in females, being 182.7 per 10^5^ in males and 186.0 per 10^5^ in females in the period from 2003–2008. Fig 2 shows the age-specific incidence for all cancers in the three time periods by sex and the trends in incidence of the four most frequent tumors in males and females. In males, the most common cancers along the study period (in terms of ASRs) were those of the prostate, Kaposi’s sarcoma (KS), the liver and the esophagus (Table 1; Fig 2C). Prostate cancer showed the most dramatic increase over the whole study period (AAPC +11.3%; 95% CI: 9.7–13.0), with an ASR of 61.7 per 10^5^ in 2003–2008 (Table 1). During the study period the increase in the incidence of prostate cancer was greater in subjects over 60 years of age compared to younger subjects (35.1 per 10^5^ versus 3.9 per 10^5^) (Fig 3A). The age of patients with cancer of the liver varied widely, with 78.1% diagnosed in patients younger than 60 years of age (Fig 3B).

**Table 1:** Age-standardized incidence rates (per 100,000) during three time periods and the average annual percentage change in age-standardized rates in the period from 1991–2008.

		MALES				FEMALES			
		Age-standardized rate per 100,000			Annual percentage change (95% confidence interval)	Age-standardized rate per 100,000			Annual percentage change (95% confidence interval)
Site	ICD-10	1991–1996	1997–2002	2003–2008		1991–1996	1997–2002	2003–2008	
Lip, oral cavity, pharynx	C00-C14	4.6	4.0	5.0	0.7% (-3.5 to 4.9)	2.2	2.4	4.5	5.5% (0.3 to 10.7)
Esophagus	C15	4.7	6.3	8.7	4.7% (1.2 to 8.2)	3.8	5.0	9.9	8.0% (4.3 to 11.8)
Stomach	C16	2.2	1.6	1.6	-3.7% (-10.4 to 3.1)	0.8	0.6	0.9	0.1% (-10.5 to 10.8)
Colon-rectum	C18-C21	3.1	3.1	6.3	6.8% (2.2 to 11.3)	2.3	2.6	2.7	1.9% (-3.7 to 7.5)
Liver	C22	18.9	9.8	14.1	-1.6% (-4 to 0.7)	7.6	4.4	8.3	2.4% (-1.1 to 5.8)
Pancreas	C25	1.9	0.8	0.4	-8.2% (-19.8 to 3.4)	0.6	1.2	0.3	-5.6% (-19.7 to 8.6)
Trachea, bronchus, lung	C33-34	6.6	3.2	2.6	-6.1% (-10.6 to -1.6)	2.3	0.9	1.7	-2.1% (-9.1 to 4.8)
Melanoma of skin	C43	1.1	1.5	1.7	3.3% (-4.2 to 10.9)	0.7	1.3	3.3	10.6% (2.5 to 18.7)
Kaposi’s sarcoma	C46	5.6	12.1	25.0	11.2% (8.6 to 13.8)	1.6	4.9	12.0	15.4% (11.1 to 19.6)
Other connective and soft tissue	C49	1.5	1.9	4.4	9.6% (3.6 to 15.5)	1.2	1.3	3.1	8.1% (1.1 to 15.2)
Breast	C50	0.3	0.8	1.2	4.8% (-5.4 to 15)	13.7	12.8	26.2	6.5% (4.3 to 8.7)
Uterine cervix	C53					34.3	43.3	62.0	4.7% (3.4 to 6)
Uterine corpus	C54					2.0	3.4	3.9	4.9% (-0.3 to 10.1)
Ovary	C56					1.8	3.0	2.9	4.1% (-1.6 to 9.7)
Penis	C60	2.2	3.1	3.6	2.9% (-2.1 to 7.9)				
Prostate	C61	17.4	28.2	61.7	11.3% (9.7 to 13)				
Kidney	C64	0.6	0.5	0.9	4.9% (-5.8 to 15.5)	0.5	0.8	1.3	8.3% (-1.7 to 18.4)
Bladder	C67	5.8	5.1	4.0	-2.7% (-6.7 to 1.3)	7.2	4.2	2.9	-7.0% (-11.2 to -2.7)
Eye	C69	1.4	1.8	4.7	10.9% (4.9 to 16.9)	1.3	1.9	6.3	13.1% (7.4 to 18.8)
Thyroid	C73	0.2	0.5	0.6	5.3% (-9.5 to 20)	1.1	1.2	3.2	8.7% (1.2 to 16.3)
Hodgkin’s disease	C81	0.3	0.7	1.4	-2.6% (-5.1 to -0.1)	0.6	0.2	0.7	3.5% (-0.2 to 7.2)
Non-Hodgkin’s lymphoma	C82-C85-C96	3.8	3.9	7.7	6.4% (2.3 to 10.5)	2.1	2.8	6.0	9.0% (4 to 14)
Other	CX	20.1	17.6	28.1	3.8% (1.9 to 5.8)	14.9	16.1	23.9	3.5% (1.4 to 5.6)

In females, the most frequent cancers over the whole study period were cancer of the uterine cervix, breast, KS and the liver (Table 1; Fig 2D). Cancer of the uterine cervix increased along the study period (AAPC + 4.7%; 95% CI: 3.4–6) with an ASR of 62.0 per 10^5^ in 2003–2008 (Table 1). The increases in the incidence of both cancer of the uterine cervix and the breast tended to be higher in women older than 40 years of age (41.9 per 10^5^ versus 6.3 per 10^5^ for cancer of the uterine cervix and 16.3 per 10^5^ versus 2.1 per 10^5^ for breast cancer) (Fig 3C and 3D). The incidence of KS was shown to markedly increase along the study period in both gender groups and in all age groups (Fig 3E and 3F).

Among children (0–14 years) 738 cancers were registered during the 18-year study period. Of the 738 pediatric tumors 420 (56.9%) occurred in males, 311 (42.1%) in females and in 7 cases the sex was not recorded. The most common tumors were non-Hodgkin’s lymphomas (32.5%) and KS (13.4%). Within the group of non-Hodgkin’s lymphoma, 48.9% (133/272) were Burkitt’s lymphoma. The incidence of non-Hodgkin’s lymphoma increased from 3.6 per 10^6^ in 1991–1996 to 20 per 10^6^ in 2003–2008, and that of KS rose from 0.4 per 10^6^ in 1991–1996 to 11.0 per 10^6^ in 2003–2008. The mean age of children with Burkitt’s lymphoma remained constant throughout the study period (6.8–6.6 years).

## Discussion

This study is one of the few reports providing solid data on cancer trends in sub-Saharan Africa over a period of 18-years. Moreover, a major strength of this report is that data from the same area had been previously published in Mozambique from the period from 1956 to 1961, thus providing the unique opportunity to compare the trends in cancer for the same area over a long period covering almost 50 years. During this period major socio-economic and behavioral changes have occurred in sub-Saharan Africa[4,6].

Cancer of the uterine cervix was found to be the most common cancer in women during the whole study period with the incidence increasing at an average of 4.7% per year, being highest at the end of the study period (ASR 62.0 per 10^5^ in 2003–2008). These results are in agreement with reports showing that cancer of the uterine cervix is the most common cancer in women in sub-Saharan Africa, with the highest incidence in eastern and southern Africa[4,7,19]. The reasons for these changes are not immediately clear. The social disruption caused by the civil war in the 1970s and 1980s may have favored the spread of human papillomavirus (HPV) infection (as well as other sexually transmitted diseases), leading to an increased risk of cancer of the uterine cervix. Increase in the awareness of the disease and improvements in the diagnostic capacity may have also played a role. Indeed, HPV infection, the cause of cancer of the uterine cervix has been shown to be highly prevalent in Mozambique with over 50% of the women being positive at 20 years of age and 20–30% of those over 30 years of age [20–22]. As in other low-income countries, the high prevalence of cancer of the uterine cervix may be explained by the difficulties in carrying out secondary prevention programs due to limited resources. On the other hand, the high prevalence of HIV infection in Mozambique has been proposed as the most likely explanation for the increase in the incidence in cancer of the uterine cervix observed in several sub-Saharan countries. According to the UNAIDS Global Report, the prevalence of HIV in Mozambique in people aged 15–49 was 9% in 2001[9], but was as high as 45% in women aged 28–47 in a recent community-based study in a rural district of Maputo province[23]. However, although linkage studies of HIV/AIDS and cancer registries have indicated a 2- to 22-fold increase in cancer of the uterine cervix in HIV-positive compared with HIV-negative women[24], the reasons for these changes are not completely understood. Moreover, although cancer of the uterine cervix is considered to be an AIDS-defining condition, it is not clear whether the association is simply due to the increased prevalence of infection with oncogenic HPV, as suggested by a number of studies[25,26].

Prostate cancer was found to be the most common cancer among men, with a significant increase in its incidence (11.3% annually on average) over the 18-year study period. Prostate cancer was rarely diagnosed in sub-Saharan Africa in the 1950s and 1960s[27,28], whereas during the period from 2003–2008 the ASR (61.7 per 10^5^) was one of the highest observed in Africa[5,8,29], with the greatest increase being observed in men of 60 years or over. Interestingly, prostate cancer is a major malignancy among men of African descent throughout the world[30] and some estimations indicate that it will become the most frequent tumor in sub-Saharan Africa. The rise in prostate cancer in Mozambique is unlikely to be due to screening programs based on prostate-specific antigen testing, although it is possible that increased awareness, a greater readiness to perform prostatectomy for urinary symptoms in elderly men and histological examination of operative biopsies might explain the increase observed in its incidence.

The incidence of breast cancer was very low in Mozambique in the 1950s[27]. Nonetheless, although the absolute incidence rate (ASR of 26.2 per 10^5^ in 2003–2008) remains relatively low by global standards[1,3,5], the incidence of breast cancer rose at a high rate annually (6.5%) during the study period, with the greatest change occurring in postmenopausal women (Fig 3D). Part of this increase may be related to an adoption of western habits especially among the urban population, leading to lower fertility rates and to a higher frequency of overweight and obesity. Although there is no information on the extent of such changes in the Maputo population, census data suggest that urban dwellers, and those with higher educational levels, have a lower than average fertility rate[14,15]; however, it is unlikely that changes in these parameters may be the only explanation for such as a large increase in the incidence of breast cancer over the study period.

All cancers related to HIV infection (KS, SCC of the conjunctiva[31,32] and non-Hodgkin’s lymphoma) showed marked annual average percentage increases (11.2% in men and 15.4% in women for KS, 10.9% in men and 13.1% in women for cancer of the conjunctiva and 6.4% in men and 9.0% in women for non-Hodgkin’s lymphoma). This is in keeping with the marked increase in the prevalence of HIV infection, which in some parts of the country has reached up to 45% among women aged 28–47 years[23]. These data are in contrast with the fall in the incidence of HIV prevalence and KS observed since the early 1990s in other sub-Saharan countries such as Uganda [7]. No changes were observed in the age-specific incidence of KS over time with the availability of antiretroviral therapy.

Different trends were observed in cancers traditionally considered to be frequent in Eastern and Southern Africa in the 1950s[27]. The incidence of liver cancer showed a decrease in men and a moderate increase in women during the study period (-1.6% and +2.4% in males and females respectively). However, in comparison with the 1950’s a marked decrease in the incidence of liver cancer has been observed, despite the relatively high rate of chronic hepatitis B and aflatoxin contamination of foodstuffs [33]. In spite of the viral etiology of liver cancer, the immunosuppression caused by HIV seems to have had little or no effect on its incidence[34]. Cancer of the urinary bladder was found to be the second most frequent cancer in males and the third in females in the period 1956–1961 with most of the cancers being SCC[27], a histological variety closely associated with urinary schistosomiasis infection[35]. Interestingly, urinary bladder cancer has shown a significant decrease, which may be related to the decrease in the prevalence of schistosomiasis recently reported in southern Mozambique[36].

Esophageal cancer, which was an infrequent cancer in the 1950s[27] showed a marked increase along the study period in both sexes. The reasons for this are not well understood. Although increased alcohol intake might play a role, other factors such as dietary deficiencies and mycotoxins have been investigated as a cause of the relatively high rates of esophageal cancer in Eastern and Southern Africa[37].

Tumors of the large bowel were relatively rare, although marked increases were observed in both men and women. With regard to stomach cancer, though the prevalence of *Helicobacter pylori* infection in adults in Maputo was high in patients with gastritis[38], this cancer was infrequent and showed no changes in incidence during the study period.

Among children the incidence of both KS and non-Hodgkin’s lymphoma (predominantly Burkitt’s lymphoma) significantly increased during the study period. This differs with the decrease in the incidence of this cancer observed in other geographical areas of sub-Saharan Africa as a result of the availability of anti-retrovirals[7,8].

A major strength of our study is that since 1991 the MCH has received virtually all the specimens from the city of Maputo during the study period and registered all the cases of cancer based on pathological verification of the diagnosis. Special efforts have been made to maximize the completeness of registration and ensure the quality of data. However, it cannot be ruled out that improvement in health service coverage in the country might have contributed to the increase of observed incidence of some cancers. In order to indicate the completeness of the registry[39], we compared the incidence rate in different populations, and the observed similarity of these findings to those published for the period of 1991–1996 in the Kyadondo County in Uganda support the accuracy of the current results[7]. Moreover, data from the 2012 International Agency for Research on Cancer (IARC) report in Mozambique and neighboring countries such as South Africa are in keeping with our results [5]. The ASR on cancer of the uterine cervix in Mozambique was 65 per 10^5^ according to the IARC report, which is similar to the 62 per 10^5^ found in the last period of the study. A significant limitation of our study is that cases of leukemia and those with only a clinical diagnosis were not registered during this period. Another limitation is that statistics in Mozambique, as in other African countries, are very sparse because of the lack of or the unreliability of data registration. Thus, the real incidence of cancer might be underestimated.

As has occurred in much of urban Africa, the lifestyles of the population of Maputo are changing rapidly with the changes in the population from one comprising relatively recent immigrants from villages, to that of second generation inhabitants, engaged in wage-earning or informal economy, and the purchasing of foodstuffs and other necessities, rather than producing themselves[4,6]. This demographic transition is accompanied by familiar trends in patterns of health and illness. It is therefore not surprising to note a steady increase in the incidence of cancer.

## Conclusions

In conclusion, the ongoing surveillance system provided by the cancer registry in this predominantly urban Southern African population has provided a picture of the evolution of cancer in modern sub-Saharan Africa. Interestingly, there is a predominance of cancers of demonstrated infectious etiology, such as HPV-associated cancer of the uterine cervix and other AIDS-related cancers (Kaposi’s sarcoma, cancer of the conjunctiva and non-Hodgkin’s lymphomas) mirroring the increase in the HIV-AIDS epidemic in the country. To date, there is little evidence of the onset of the epidemic of tobacco-related cancer which has been such an important feature of the cancer profile in economically developed countries. These findings are of relevance to guide cancer prevention policies in Mozambique and countries of similar characteristics.
